# An Interactive Health Communication Application for Supporting Parents Managing Childhood Long-Term Conditions: Outcomes of a Randomized Controlled Feasibility Trial

**DOI:** 10.2196/resprot.3716

**Published:** 2014-12-03

**Authors:** Veronica M Swallow, Kathleen Knafl, Sheila Santacroce, Malcolm Campbell, Andrew G Hall, Trish Smith, Ian Carolan

**Affiliations:** ^1^School of Nursing, Midwifery and Social WorkFaculty of Medical and Human SciencesUniversity of ManchesterManchesterUnited Kingdom; ^2^School of NursingUniversity of North Carolina at Chapel HillChapel Hill, NCUnited States; ^3^NephrologyDept of Paediatric NephrologyRoyal Manchester Children's HospitalManchesterUnited Kingdom; ^4^Sefton Local AuthorityLiverpoolUnited Kingdom

**Keywords:** child, chronic condition, chronic kidney disease, CKD, family, feasibility, interactive health communication application, online, long-term condition, parent, randomized controlled trial

## Abstract

**Background:**

Families living with chronic or long-term conditions such as chronic kidney disease (CKD), stages 3-5, face multiple challenges and respond to these challenges in various ways. Some families adapt well while others struggle, and family response to a condition is closely related to outcome. With families and professionals, we developed a novel condition-specific interactive health communication app to improve parents’ management ability—the online parent information and support (OPIS) program. OPIS consists of a comprehensive mix of clinical caregiving and psychosocial information and support.

**Objective:**

The purpose of this study was to (1) assess feasibility of a future full-scale randomized controlled trial (RCT) of OPIS in terms of recruitment and retention, data collection procedures, and psychometric performance of the study measures in the target population, and (2) investigate trends in change in outcome measures in a small-scale RCT in parents of children with CKD stages 3-5.

**Methods:**

Parents were recruited from a pediatric nephrology clinic and randomly assigned to one of two treatment groups: usual support for home-based clinical caregiving (control) or usual support plus password-protected access to OPIS for 20 weeks (intervention). Both groups completed study measures at study entry and exit. We assessed feasibility descriptively in terms of recruitment and retention rates overall; assessed recruitment, retention, and uptake of the intervention between groups; and compared family condition management, empowerment to deliver care, and fathers’ involvement between groups.

**Results:**

We recruited 55 parents of 39 children (42% of eligible families). Of those, about three-quarters of intervention group parents (19/26, 73%) and control group parents (22/29, 76%) were retained through completion of 20-week data collection. The overall retention rate was 41/55 (75%). The 41 parents completing the trial were asked to respond to the same 10 questionnaire scales at both baseline and 20 weeks later; 10 scores were missing at baseline and nine were missing at 20 weeks. Site user statistics provided evidence that all intervention group parents accessed OPIS. Analysis found that intervention group parents showed a greater improvement in perceived competence to manage their child’s condition compared to control group parents: adjusted mean Family Management Measure (FaMM) Condition Management Ability Scale intervention group 44.5 versus control group 41.9, difference 2.6, 95% CI -1.6 to 6.7. Differences between the groups in the FaMM Family Life Difficulty Scale (39.9 vs 36.3, difference 3.7, 95% CI -4.9 to 12.2) appeared to agree with a qualitative observation that OPIS helped parents achieve understanding and maintain awareness of the impact of their child’s condition.

**Conclusions:**

A full-scale RCT of the effectiveness of OPIS is feasible. OPIS has the potential to beneficially affect self-reported outcomes, including parents’ perceived competence to manage home-based clinical care for children with CKD stage 3-5. Our design and methodology can be transferred to the management of other childhood conditions.

**Trial Registration:**

International Standard Randomized Controlled Trial Number (ISRCTN): 84283190; http://www.controlled-trials.com/ISRCTN84283190 (Archived by WebCite at http://www.webcitation.org/6TuPdrXTF).

## Introduction

Children and young people (children) aged 0-19 with conditions such as chronic kidney disease (CKD), stages 3-5, often require treatments at home, which can be complex and intrusive. Research into long-term or chronic childhood (hereafter referred to as chronic) conditions helps us understand how families manage the child’s condition at home with remote support from multidisciplinary teams (MDTs) [1-5]. CKD, a complex set of disorders with a wide range of primary causes and complications has an unpredictable course. Parents’ home-based clinical management responsibilities include fluctuating levels of monitoring and intervention, which can be complicated, intrusive, and require skilled work by families. This skilled work can present extensive challenges for parents and be difficult for them to maintain, especially if they do not possess the comprehension needed to understand instructions. For example, parents may need to collect or test urine samples; understand clinically indicated investigations such as laboratory and imaging studies; administer medications; conduct gastrostomy or naso-gastric tube feeds; set up and run peritoneal dialysis; carefully monitor diet and fluids; and recognize and act on subtle but significant clinical changes in their child. Additionally, parents need to communicate effectively with staff and coordinate many aspects of personal and clinical care while supporting their child, promoting child development, and maintaining normal family life. Parents’ failure to become competent at clinical management could lead to non-adherence to treatment regimens, inability to recognize and respond to significant clinical changes, and negative clinical outcomes for the child such as undetected urinary tract infection, which can further damage the kidneys and impair kidney function [2]. In many children, CKD progresses from stage 3 when they require careful monitoring and occasional medications or clinical investigations, to stage 5 when they require renal replacement therapies such as dialysis or transplantation [6] (Table 1).

**Table 1:** Chronic kidney disease stages 3-5 (adapted from the Renal Association).

CKD stage (GFR^a^)	Clinical criteria	Clinical management
3a (35-59%)	Moderately reduced kidney function	Observation, control of blood pressure, and risk factors for progression to stage 4
3b (30-34%)		
4 (15-29%)	Severely reduced kidney function	Planning for end stage renal failure
5 (<15% or on dialysis)	Very severely reduced kidney function or end-stage renal failure	Treatment choices (renal replacement therapies)

^a^GFR=estimated Glomular Filtration Rate.

Families respond in various ways to chronic conditions. Some adapt well to clinical management responsibilities and are able to develop a sense of control over their lives while others struggle to do so. Family response to chronic conditions is closely related to children’s clinical outcomes, and non-adherence to prescribed treatments is the primary cause of treatment failure in conditions such as CKD [7-9]. The burden of condition management generally lies with parents and other caregivers rather than the child. A recent systematic review of qualitative studies of parents’ views on treatment non-adherence in various medical conditions found caregivers worked hard to retain a sense of control by dealing with challenges such as the child’s resistance to treatments. Nevertheless, strict treatment adherence, which is expected by health professionals, could threaten parents’ priorities around preserving family relationships and providing a “normal family life” [10,11].

A sense of control has also been associated with the notion of empowerment in pediatric care [12]. Parents want to be empowered to competently deliver clinical care, to recognize and respond appropriately to changes in the child’s condition, and to communicate effectively with health professionals about condition management and to relatives, friends, and teachers about the implications of the condition for the child [1,13-16]. Moreover, professionals wish to empower parents, and strategies to help them do this include promoting equal relationships, critical reflection and advocacy, focusing on strengths, supporting active participation and decision-making, providing information, and developing skills [17].

It is important that children with chronic conditions are cared for in ways that minimize emotional trauma and assist in their recovery, and that such ways of delivering care are investigated to see if they are effective [18,19]. In a recent ethnographic study of interactions between fathers, mothers, and professionals during shared care of CKD, it was observed that over time, professionals developed a shared repertoire of tools and artefacts (such as diagrams, anatomically correct dolls, booklets) to support their communications with parents, and that these tools and artefacts helped some parents and professionals to accomplish common ground [4]. Although both parents are often involved in caring for children with chronic conditions, fathers’ views tend to be underrepresented in the health care literature [20]. However, evidence is now emerging about the extent of the fathers’ involvement and the value of fathers’ involvement for children, families, and fathers themselves [20-22].

In addition, family responses to chronic condition management can be affected by individuals’ health literacy skills (ie, the ability to comprehend health information) [23]. Parents are increasingly likely to search the Internet for information and support to supplement the guidance they receive from health professionals. However, few interactive, condition-specific, evidence-based online resources are available. Where online resources do exist, they are often based on myth and hearsay [15], and parents with poor literacy levels may be unable to discriminate between high and low quality information and may not be confident in using the Internet [24,25]. Therefore, rigorously developed and evaluated online resources that meet parents’ and professionals’ needs and preferences are required.

A Cochrane Review [16] shows the use of interactive health communication applications (IHCAs)—computer-based, usually Web-based, information packages for patients/carers that combine health information with social support, decision support, or behavior change support— has positive effects on users. IHCA users tend to become more knowledgeable and perceive higher levels of social support than non-users. Patients who have access to IHCAs either themselves or via a caregiver/parent might have improved behavioral and clinical outcomes compared to non-users, and IHCAs are more likely than not to have a positive effect on users’ management ability and self-efficacy. Therefore, more high-quality studies are recommended to determine the best type of and best way to deliver IHCAs, and to establish how IHCAs affect different groups of people with chronic illness [16].

**Figure 1:**
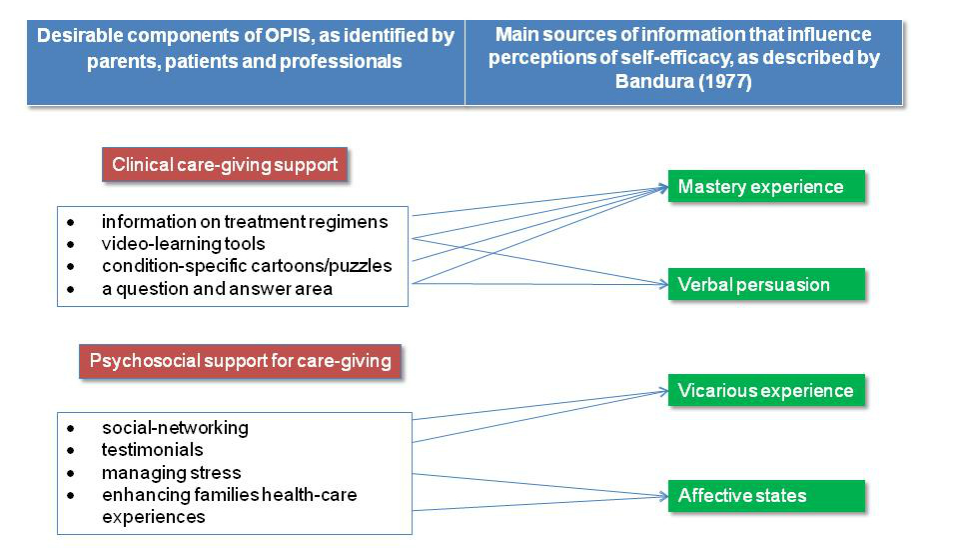
Online Parent Empowerment Model in CKD management [28].

The current study forms part of a phased-approach to development and evaluation of a complex intervention [26], an IHCA for parents of children with CKD stage 3-5. This study was framed by Bandura’s concept of self-efficacy, which provides a basis for understanding personal motivation, well-being, and feelings of personal accomplishment in situations that are cognitively, behaviorally, and emotionally challenging [27]. To help parents develop self-efficacy for managing their child’s CKD, we first developed an online parent empowerment model in CKD management [28]. The main sources of information that influence perceptions of self-efficacy (mastery experience, vicarious experience, verbal persuasion or similar sources of social influences, and affective states [27]), when integrated with the two main components of our Online Parent Information and Support (OPIS) app, as required by parents and professionals (clinical care-giving support and psychosocial support for care-giving) resulted in the model (Figure 1).

We developed an IHCA, the OPIS application, in collaboration with families and health professionals. The OPIS comprises clinical care-giving support (information on treatment regimens, video-learning tools of MDT professionals explaining how to undertake clinical procedures at home*,* condition-specific cartoons/puzzles, and a question and answer area) and psychosocial support for caregiving (social networking, testimonials from other parents of children with CKD, and advice on managing stress) [11,28].

We implemented and assessed OPIS for feasibility in the kidney unit of a large children’s hospital in the north of England [28,29]. In an earlier report, we provided evidence that parents found OPIS to be very usable and acceptable, and implementation into standard practice was shown to be feasible [30]. In fact, 93% of users reported that OPIS was easy to use and therefore, there was confidence that the design and technology of the ICHA was not a barrier to its use. Qualitative suggestions by parents included refinement of OPIS components, enabling personalization of OPIS functionalities, and proactive endorsements of OPIS by professionals [28].

The purpose of this paper is to build on our previous report [30] by presenting the results of a study that addressed two objectives: (1) to assess the feasibility of a future full-scale randomized controlled trial (RCT) of OPIS in terms of recruitment and retention of mothers and fathers, data collection procedures, and psychometric performance of the study measures in the target population, and (2) to investigate trends in change in outcome measures in a small-scale feasibility RCT in parents of children with CKD stages 3-5.

The results reported here will inform the development and implementation of a future RCT that will be sufficiently powered to detect significant change in outcomes attributable to OPIS. To our knowledge, OPIS is the first IHCA to have been rigorously developed with families living with CKD and health professionals, and then tested with parents.

## Methods

### Study Design

To achieve the stated objectives, we undertook a small-scale study that used a two-group RCT design with data collected at two points in time: entry to the study at baseline and 20 weeks later (ISRCTN: 84283190). Approval to conduct the study was obtained from the National Health Service (NHS) Research Ethics Committee (REC) (Reference: 11/N/W/0268) and the NHS Trust Research and Development department. No incentives were offered to parents for enrolling in the study.

### Treatment Conditions

Parents who provided written informed consent were randomly assigned to one of two treatment conditions: (1) usual support involving discussions with members of the MDT when the child was an in- or out-patient, and for children with stage 5 CKD, home visits from a specialist nurse to teach or reinforce clinical skills, as required (control group), or (2) usual support plus password-protected access to OPIS for 20 weeks, which allowed sufficient time for participants to become familiar with OPIS (intervention group). Children at stage 5 CKD require peritoneal dialysis treatment; the specialist nurse teaches the child and family how to perform this treatment over a series of home visits until they are considered competent to perform it on their own. Future home visits follow to ensure the treatment is being performed correctly. Other clinical skills including giving injections, and managing nasogastric or gastrostomy feeds would also be taught at home with ongoing support to ensure competency.

**Figure 2:**
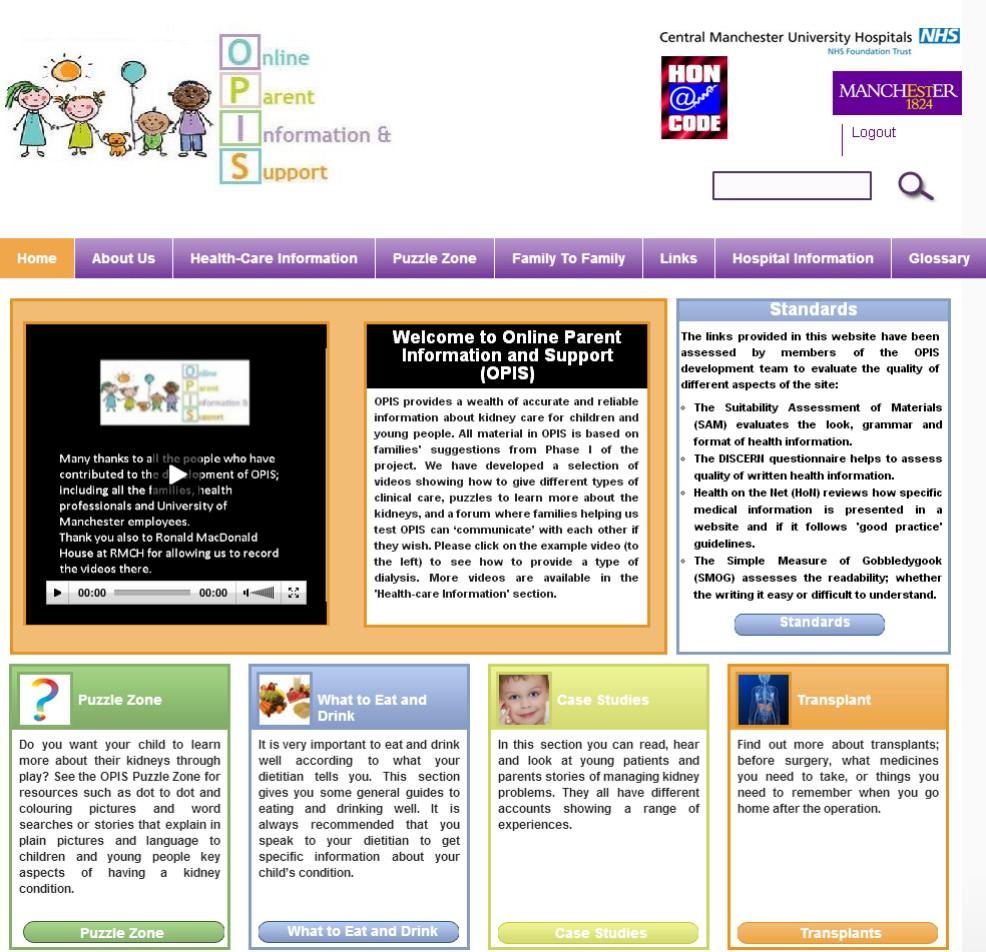
Screenshot of OPIS homepage.

OPIS was housed on a university Web server and accessible to intervention group parents via their personal computers, mobile phones, tablets, or smartphones. A screenshot of one view of OPIS is shown in Figure 2.

OPIS is Health on the Net (HON) certified (HONConduct443339) [31]. The HON certificate serves as a guarantee that OPIS complies with and pledges to honor the eight principles of the Code of Conduct developed by the HON Foundation: Authority, Complementarity, Confidentiality, Attribution, Justifiability, Transparency, Financial Disclosure, and Advertising. More detailed description and discussion of the development, implementation, usage, and assessment of acceptability and usability of OPIS have been published elsewhere [30,32].

### Sample

Parents were considered eligible for this study if their child aged 0-19 years was receiving care at the study site, they had not participated in the development of OPIS and they had access to the Internet via a personal computer or mobile device. Eligible parents were notified of the study by a member of the MDT. Interested parents were referred to the researcher appointed to manage the project and collect/analyze data who then explained study requirements, answered any questions, and obtained written adult consent. As fathers and mothers may have differing information and support needs when their child has a chronic condition [13,20,22,33-36], both parents were invited to participate if they shared the clinical caring role. After baseline data collection, participating parents were then randomly allocated to either the control group or the intervention group at the family level. That is, if two parents of an index child were enrolled in the study, both parents were allocated to the same treatment condition. One of the authors who was not involved in data collection, generated the randomized allocation sequence using nQuery Advisor 6.0, with blocks of random length in an allocation ratio of 1:1, randomized and stratified by CKD stage (3 vs 4/5) and ethnicity (white/black vs South Asian) of the child. Clinical care needs can vary according to CKD stage (Figure 1), and CKD prevalence is higher in UK South Asian groups relative to their representativeness in the overall UK population [37]; thus, we aimed to distribute disease stage and parent race/ethnicity equally between the two treatment groups despite the small scale of the study. The allocation sequence was concealed from parents and the researcher using sequentially numbered opaque envelopes containing the allocation code, which were prepared by a person not otherwise involved in the study. However, blinding parents to whether or not they had been assigned to receive the intervention was not possible. While our working benchmark for feasibility was to have 30 parents in each group by end trial for this pilot/feasibility study [38], we acknowledged from the start that we would be limited by the number of eligible children under care at the study site at that time.

### Data Collection Procedure

Data were collected between September 2012 and September 2013. Measurements were conducted before parents were randomized. Those in the intervention group received a username and password to enable OPIS access, and after 20 weeks at which point their access to OPIS ceased. Data collection took place at a time/place convenient to the parents, either in the family home or a quiet area in the hospital; one interview was conducted by telephone at the parent’s request [39]. Evidence is emerging of the persuasive practices of some parents to engage their families in research, which underlines the importance of accessing all potential participants directly, and the importance of sensitization to interactions between family members when engaging in research [37]. Based on this emerging evidence, including our own experience of purposefully recruiting mothers, fathers, and children separately to research (eg, [13,33]), in families where both parents participated in the current study, we ensured that they completed the measures independently of each other. That is, they completed the measures in separate rooms and were not allowed to discuss the measures until each had completed their measure.

### Study Measures

At baseline, we used an investigator-devised form to collect background data (child age, sex, postal code, CKD stage at study entry/exit; parent age, sex, race/ethnicity, language, educational achievement, socioeconomic status of neighborhood based on postcode, ethnicity, and clinical care experience). At both baseline and 20 weeks, we administered a set of standardized measures in the following order: the Rapid Estimate for Adult Literacy in Medicine (REALM) [40], the Family Management Measure (FaMM) [8], the Service System Subscale of the Family Empowerment Scale (FES) [41], and the Dads Active Disease Support Scale (DADS) [42]. At baseline, background data were collected first followed by administration of the standardized measures in the order stated. At 20 weeks, the standardized measures were re-administered in the order stated. Completion of the study measures took 40-55 minutes overall.

Parent health literacy was measured using the REALM. The purpose of this was to determine if parents were likely to need help with self-administration of the outcome measures. This assessment requires the parent to read aloud a list of 66 generic clinical words (such as “fat” or “impetigo’) arranged in increasing order of difficulty. The score is calculated by awarding one point for each correctly pronounced word and nil for each mispronounced or skipped word. A score of 59 or less indicates low health literacy while a score of 60 or more indicates adequate health literacy. The REALM has face, criterion, and construct validity for use as a health literacy screening tool in the United Kingdom [40].

Parent management ability was measured using the FaMM. The FaMM was developed to measure how families manage caring for a chronic condition and the extent to which they incorporate management into family life. The FaMM has 53 items overall, with 45 items for all parents and eight additional items for partnered parents only. Items are scored from 1-5, meaning strongly disagree to strongly agree. There are five summated scales for all parents measuring the dimensions of Child’s Daily Life, Condition Management Ability, Condition Management Effort, Family Life Difficulty, and View of Condition Impact as well as a sixth scale only for partnered parents measuring the dimension of Parental Mutuality.

The FaMM Condition Management Ability Scale (12 items) addresses parents’ perceptions of their competence at taking care of the child’s condition. Because the intent of the OPIS intervention was to enhance parents’ ability to manage their child’s CKD, we were especially interested in the Condition Management Ability scale of the FaMM. Higher values mean parents view themselves as more capable of managing the condition. Example items that help to illustrate the concept and its domains include (1) “We have some definite ideas about how to help our child live with the condition”, and (2) “We have not been able to develop a routine for taking care of our child’s condition” [8].

Parent empowerment was measured using the Service System Subscale of the FES that explores parents’ relationships with health professionals and parents’ level of comfort in asking questions and voicing their opinions [41]. The FES measures caregivers’ beliefs and confidence regarding the services the child needs, their initiative in obtaining these services and making sure that the professionals understand and respect their opinions regarding what the child needs, their knowledge and understanding of services, and their positive attitudes about their ability to obtain and claim the services the child needs. The items are scored in the same direction and higher scores indicate relatively more empowerment in a specific area. The FES has robust psychometric properties and therefore has value in assessing the empowerment status of families.

Father support for managing the child’s CKD was measured by the DADS, a 24-item Likert-type scale with separate forms for mothers and fathers. The DADS was developed to assess the support offered by fathers, and mothers’ perceptions of the quality of that support. The results of confirmatory factor analysis provide support for the construct validity of the DADS, and two factors (amount and helpfulness of fathers’ involvement) best accounted for participants’ responses [42]. The DADS has been used in other studies of family response to childhood chronic conditions and the level of reliability was acceptable [21]. To our knowledge, our study represents the first use of the DADS, the FaMM, and the FES, all of which were developed in North America, with parents in the United Kingdom. However, we did not observe any difficulties when parents were completing these measures about the interpretation and understanding of items and response ratings.

### Analysis

Data were analyzed using IBM SPSS Statistics Version 20. Participants who dropped out were not contacted further in keeping with REC approval, and the data they provided were compared with those of participants who completed the study. Scores on the outcome measures were calculated and missing values on items handled according to the methods prescribed by the developers. Consistent with the nature of the study and small sample size, our post-intervention analyses should be interpreted conservatively. Intraclass correlation coefficients (ICC) were estimated for each outcome measure to assess the level of within-family variation, and model performance was measured by estimating the square of Pearson’s correlation between actual and predicted values. Confidence intervals were estimated for recruitment and retention rates. The internal consistency of all outcome measures was estimated using Cronbach alpha at baseline across the two groups combined for those completing the study, as the outcomes were measured on a population that had not been previously assessed.

Analysis of trends in change in outcome measures from 20 weeks was adjusted for baseline scores using linear mixed models to allow for having more than one parent participating in a family with adjustments for stratification by CKD stage (3 vs 4/5) and ethnicity (white/black vs South Asian). Confidence intervals and effect sizes for the adjusted differences in means at 20 weeks were estimated—the aim in this feasibility study being to inform sample size estimates for a future full-scale RCT rather than to detect significant differences [38].

## Results

### Objective 1: Feasibility of Recruitment and Retention and Application Usage

**Figure 3:**
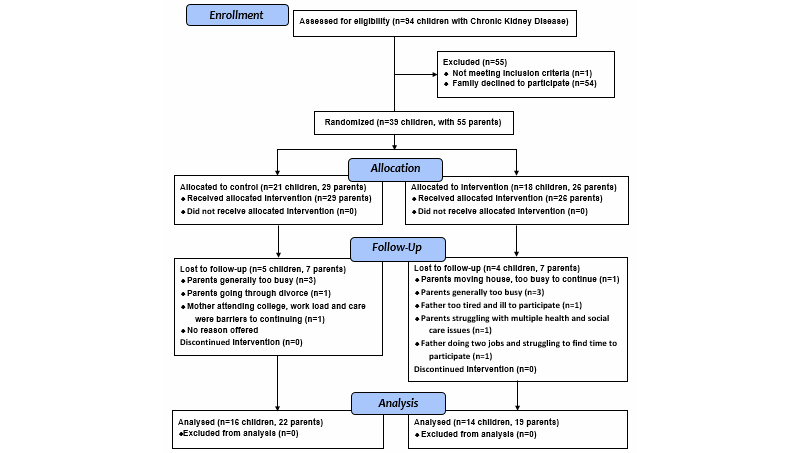
CONSORT diagram showing participant flow through study.

A total of 94 eligible children were identified at baseline. A CONSORT diagram (Figure 3) describes the recruitment and retention of parents through the phases of the feasibility RCT. In total, 39 index children with 55 parents (42% of eligible families invited, 95% CI 32-52) participated. Data at 20 weeks were provided by approximately three-quarters of parents in the control group (22/29, 76%; 95% CI 58-88) and in the intervention group (19/26, 73% of those participating; 95% CI 54-85).

At baseline, the two groups were balanced in terms of the stratification variable ethnicity (white/black/Afro-Caribbean vs South Asian) but not CKD Stage (3 vs 4/5). No parent who recorded a REALM score of less than 60 (ie, low level of health literacy) at baseline withdrew from the study before trial end. Table 2 summarizes parent and child characteristics by group allocation at baseline for those completing the trial.

A quarter of participants (14/55, 25%) were not retained through trial end, including seven from each treatment group. At enrollment, participants were told they could withdraw from the study at any time without providing a reason. However, most parents who withdrew apologized and volunteered a reason (see Figure 2). Control group parents who withdrew tended to be slightly younger than those who were retained (mean ages 37.0 vs 44.1 years); corresponding ages in the OPIS group were similar (41.4 vs 42.7 years). In both treatment groups, parents who withdrew from the study tended to have a lower socioeconomic status based on neighborhood ranking than those who were retained. In the control group, half (5/10) of those whose child had CKD stage 3 were retained through trial end, compared with the majority whose child had CKD stage 4 or 5 (17/19, 89.5%). In the intervention group, the proportions were as follows: child with CKD stage 3 (8/10, 80.0%) versus child with CKD stage 4 or 5 (11/16, 68.6%). Otherwise, characteristics of parents who were retained through trial end were similar to characteristics of those who were not.

**Table 2:** Parent and child characteristics by randomization group at baseline for those completing trial.

Characteristic		Control group	Intervention group
Participants, n		22	19
Index children, n		16	14
Parent age, mean (SD)		44.1 (8.3)	42.7 (10.3)
**Parent gender, n (%)**			
	Female	11 (50)	11 (58)
	Male	11 (50)	8 (42)
**Parent ethnicity, n (%)**			
	White European	16 (73)	16 (84)
	Afro-Caribbean	0 (0)	1 (5)
	South Asian	6 (27)	2 (11)
**Parent primary language, n (%)**			
	English	19 (86)	19 (100)
	Bengali	2 (9)	0 (0)
	Polish	1 (5)	0 (0)
Parent socioeconomic status^a^, median (range)		13,041.5 (96-28,036)	21,547 (235-29,472)
Child age in years, mean (SD)		10.2 (5.7)	9.1 (5.5)
**Child gender, n (%)**			
	Female	5 (31)	4 (29)
	Male	11 (69)	10 (71)
**Child CKD stage, n (%)**			
	Stage 3	3 (19)	6 (43)
	Stage 4	5 (31)	0 (0)
	Stage 5	8 (50)	8 (57)

^a^Postal code-based neighborhood ranking (1=highest level of deprivation to 32,482=lowest deprivation).

#### Application Usage

In total, 19 parents accessed OPIS with a mean of 23.3 visits per user (SD 20.8, range 2-64); OPIS was visited 443 times with a total of 3154 page visits. The mean duration of time spent on the site per visit was 12 minutes, 11 seconds (range 2 seconds to 58.0 minutes). Visits lasted between 10 and 30 minutes, and 88.9% (394/443) of visitors used desktop/laptop computers, 7.9% (35/443) used mobile phones, and 3.2% (14/443) used tablets. Tablet users spent the longest time on OPIS while the desktop/laptop users spent the shortest. The most common depth of visit (25.5%) entailed viewing 20 or more different screens, with parental viewing in 34.6% of these visits ranging from 9-19 different pages. The highest number of page views in 1 week was 541. There were two peaks in usage: one at the start of the study, and the other when users were notified that new research reports regarding CKD were placed in OPIS by the project team. The most popular area was “Kidney Health” followed by “Case Studies” and “What to eat/drink”. The least popular area was “FAQ”. Average time for page download was 1.8 seconds [30].

#### Qualitative Findings

During parent exit interviews, the issue of usage feasibility was frequently highlighted by parents [30,43], as indicated by the following illustrative quotations. Parents appreciated the opportunity to hear and read accounts on OPIS from other parents of children with CKD: “I think the information was pretty good on it, from the first one when I went onto it, because the main thing is you want to see how other parents are going on with it [CKD management]” [parent/071/mother].

In addition, parents found OPIS easy to access and valued the section that describes the roles and responsibilities of the different MDT members responsible for their child’s overall CKD management: “Because it’s always nice that if you go into somewhere knowing you’ve got a picture of a face with a name, you think ah, yeah, we know her” [parent/056/father].

Parents found the range of information on OPIS interesting: “I enjoyed having a good look around it, I found it interesting for somebody that’s involved in it [CKD management] as a parent” [parent/052/mother].

Furthermore, parents appreciated the links provided to other related websites that had been validated by the OPIS research team, including MDT professionals: “Really liked trustworthy links page, knowing the MDT has agreed to them, it really helps, knowing it’s the right information and not scaring you half to death!” [parent/045/mother].

The REALM required self-administration for assessment of health literacy. Parents were given the option to self-administer the other study measures or have the researcher read the questions aloud and record the parent’s response. At baseline, 96% (53/55) of the parents opted to self-administer the other measures, and for expediency some chose to do this while waiting for their child’s outpatient appointment. Parents offered two main reasons for preferring self-administration: either (1) they wanted to complete data collection as quickly as possible because of time constraints imposed by their child’s clinical demands and their own personal commitments, or (2) reading the questions themselves helped them to better understand the issues being explored and to consider their response.

Observing while parents self-administered the REALM enabled the researcher to discreetly determine whether a respondent may have difficulty completing the remaining measures without assistance. Most parents had little difficulty completing REALM, but two fathers struggled to read out several words. In these instances, the researcher adopted an encouraging and reassuring tone, explaining that our purpose was not to judge parents but to help us learn from parents how best to explain clinical terms.

Although flexibility and respect for parents’ preferences regarding place and time for data collection is important, the outpatient waiting area was not always appropriate for this purpose. For example, with the two fathers who displayed discomfort with REALM, the researcher suggested a move to a more private location nearby. However, even after the change of location these fathers both read out the words in an increasingly lower register, with a bowed head and constricted body language. In one instance, the researcher stopped REALM administration before it was completed because of the father’s profound reading difficulty and apparent discomfort. The father went on to complete the remaining measures with the researcher reading questions aloud to him.

At baseline, members of the control group had the four lowest REALM scores (10, 42, 43, and 43); a member of the OPIS group had the fifth lowest score (57). The REALM score at both baseline and 20 weeks was recorded for 82% (18/22) of parents in the control group and 95% (18/19) of parents in the intervention group. Of these parents, 86% (31/36) scored 60 or above (denoting an adequate level of health literacy) at both time points. The remaining 5 parents scored less than 60 (denoting inadequate health literacy) at both time points and 4 of these parents were in the control group.

Administering the FaMM (the measure with the next highest number of items) immediately after the REALM meant that the researcher could reassure parents that the remaining measures (FES and DADS) would not be as time consuming to complete. After completing the FaMM, all parents volunteered that it had helped them to appreciate the amount and level of clinical care they provided for their child. They also said the FaMM prompted them to reflect on issues such as their child’s education and well-being, and how they enabled their child to achieve a good quality of life. While completing the FaMM, two mothers became emotionally distressed as they recalled the burden of care management; the researcher offered to suspend the interview if it was becoming burdensome or to arrange a meeting with a member of the MDT for counseling. Both mothers declined this offer citing the therapeutic benefit for them of processing these emotions and that through participating they were making a positive contribution to future management by other families. A few parents (8/55, 15%) parents stated that some items on the FaMM (such as “It seems as if our child’s condition controls our family life” and “Our child’s condition requires frequent hospital stays”) were not appropriate to their current situation.

The Service System subscale of the FES appeared to be easily understood by parents as no clarification was requested. The DADS scale was also easily understood.

### Psychometric Performance of the Study Measures in the Target Population

Table 3 shows descriptive statistics and reliability estimates for the study measures across the total sample at baseline for the 41 parents who completed the study. Missing values were few across the 10 measures at baseline and 20 weeks. With regard to the Table 3 column entitled, “percentage relevant scored”, all 41 parents were scored on five of the six FaMM scales at both time points; the 6 parents without a score on the Parental Mutuality Scale were participating in the study as a single-parent family. However, these 6 parents may have had a parenting partner (marital status was not sought), but if there was a partner, then he/she was not a study participant. The DADS scales were also designed for 2-parent families; the same 6 parents plus another 3 and 2 parents respectively had missing values on the DADS Amount or Helpfulness scales, again at both time points. Two parents did not complete the Service System Subscale of the FES at both time points, while 5 parents did not complete the REALM at baseline and 4 did not complete it at 20 weeks.

The majority of the outcomes measures had an acceptable level of internal consistency reliability; a Cronbach alpha ≥.70 is commonly considered as being acceptable in psychosocial research. The two exceptions were the FaMM Condition Management Ability Scale (alpha=.52) and Condition Management Effort Scale (alpha=.62). In the Condition Management Ability Scale, the item “We have enough money to manage our child’s condition” was negatively correlated with eight of the other 11 items, and its correlation with the sum of the other items was -.21. Deleting this item from the scale increased Cronbach alpha from .52 to .62. Another two items, “We have some definite ideas about how to help our child live with the condition” and “We often feel unsure what to do regarding our child’s condition”, showed very little correlation (*r*<.04) with the sum of the other items, but separately deleting either of these would have raised Cronbach alpha by only .02. We decided to delete the item “We have enough money to manage our child’s condition” from the Condition Management Ability Scale, and the following analyses are based on the revised version of this scale.

Parents had not needed several items in the DADS during the previous 6 months. For example, 20 parents had not attended a support group or educational workshop about their child’s condition and had not needed to pay medical bills. Only two parents had complete entries for the Amount score. Table 4 shows descriptive statistics at baseline for the questionnaire scores by randomized group also for parents who completed the study. On the whole, mean scores of most outcome measures in the two groups tended to be similar at baseline.

**Table 3:** Descriptive statistics and reliability estimates for the study outcome measures at baseline across total sample for parents completing the trial.

Outcome measure		Items, n	Complete responses, n	Number scored, n	Percentage relevant scored, %	Mean (SD, range)	Cronbach alpha^c^
**Family Management Measure**							
	Child’s Daily Life Scale	5	39	41	100.0	17.3 (5.0, 10-25)	.72
	Condition Management Ability Scale	12	41	41	100.0	45.0 (5.9, 27-56)	.52
	Condition Management Ability Scale (revised)^a^	11	41	41	100.0	43.6 (5.4, 32-55)	.62
	Condition Management Effort Scale^b^	4	41	41	100.0	14.0 (3.6, 6 -20)	.62
	Family Life Difficulty Scale^b^	14	40	41	100.0	36.1 (12.3, 14-56)	.90
	Parental Mutuality Scale	8	35	35	100.0	33.4 (6.2,19-40)	.79
	View of Condition Impact^b^	10	38	41	100.0	30.1 (6.3,14-41)	.69
**Family Empowerment Scale**							
	Service System Subscale	12	38	39	95.1	4.2 (0.5, 3.1-5)	.85
**Dads’ Active Disease Support Scale**							
	Amount score	24	33	33	94.3	79.2 (21.2, 50.1-120.0)	.91
	Helpfulness score	24	32	34	97.1	73.0 (19.9, 33.0-112.0)	.95

^a^Excluding the contradictorily correlated item “We have enough money to manage our child’s condition”.

^b^Higher scores are undesirable.

^c^Based on complete responses for each scale.

**Table 4:** Outcome scores at baseline by randomized group for parents completing the trial.

Outcome measure		Control group (n=22)			Intervention group (n=19)		
		n	Mean (SD)	Range	n	Mean (SD)	Range
**Family Management Measure**							
	Child’s Daily Life Scale	22	17.6 (5.6)	10-25	19	17.0 (4.4)	11-25
	Condition Management Ability Scale (revised)	22	42.8 (4.8)	36-52	19	44.5 (6.0)	32-55
	Condition Management Effort Scale^a^	22	13.1 (3.9)	6-20	19	14.9 (3.0)	8-20
	Family Life Difficulty Scale^a^	22	35.3 (13.8)	14-56	19	37.0 (10.6)	15-56
	Parental Mutuality Scale	19	32.8 (6.3)	19-40	16	34.1 (6.1)	20-40
	View of Condition Impact^a^	22	30.3 (6.0)	20-41	19	29.8 (6.8)	14-40
**Family Empowerment Scale**							
	Service System Subscale	17	4.3 (0.5)	3.1-5.0	16	4.1 (0.5)	3.2-4.9
**Dads’ Active Disease Support Scale**							
	Amount score	17	84.4 (22.5)	50.7-120.0	16	73.9 (18.7)	50.1-115.2
	Helpfulness score	18	69.9 (21.1)	41.8-102.0	16	76.6 (18.4)	33.0-112.0

^a^Higher scores are undesirable.

### Objective 2: Trends in Change in Outcome Measures

Table 5 presents the estimated marginal means for the outcome measures at 20 weeks by randomized group for parents completing the trial, adjusted for baseline scores, stratification variables, and number of parents participating. The ICCs indicate the proportion of variance that can be attributed to differences between families. High values indicate more variation between families (ie, less variation between parents), while low values indicate less variation between families (ie, more variation between parents). Among those whose child’s other parent participated in the study, parent dyads showed most agreement in outcomes concerning the practical impact of their child’s condition (FaMM View of Condition Impact and Family Life Difficulty Scales). They showed least agreement on the support given by the other partner or the father (FaMM Parental Mutuality Scale and DADS Helpfulness score). While the numbers of parents involved were small, this finding requires further investigation in a fully powered trial. The most noticeable difference was in the change in DADS Helpfulness in the intervention group; mothers’ mean score for their partner increased by 15.0 (n=7) from baseline to end trial while fathers’ self-perceived mean score fell by 6.0 (n=5). Corresponding changes in the control group were a decrease of 3.5 (n=6) reported by mothers and a decrease of -0.2 reported by fathers.

**Table 5:** Estimated marginal means^a^ for outcome scores by randomized group and their differences at end trial using linear mixed model.

Outcome measure		Control group (n=22)		Intervention group (n=19)		Intervention minus Control			
		n	Mean^a^ (95% CI)	n	Mean^a^ (95% CI)	Diff (95% CI)	*P* value	*r* ^*2*^	ICC
**Family Management Measure**									
	Child’s Daily Life Scale	22	16.9 (13.8-19.6)	19	15.7 (12.8-18.7)	-0.9 (-4.3 to 2.5)	.576	.856	.568
	Condition Management Ability Scale (revised)	22	41.9 (38.5-45.4)	19	44.5 (40.9-48.1)	2.6 (-1.6 to 6.7)	.213	.823	.440
	Condition Management Effort Scale^b^	22	13.3 (11.0-15.6)	19	15.2 (12.8-17.6)	1.8 (-0.9 to 4.6)	.176	.613	.247
	Family Life Difficulty Scale^b^	22	36.3 (29.0-43.5)	19	39.9 (32.5-47.3)	3.7 (-4.9 to 12.2)	.389	.937	.778
	Parental Mutuality Scale	19	31.0 (27.7-34.3)	16	34.8 (31.4-38.2)	3.8 (-0.3 to 7.9)	.066	.421	.138
	View of Condition Impact^b^	22	29.9 (26.1-33.7)	19	30.6 (26.8-34.4)	0.7 (-3.8 to 5.1)	.763	.953	.829
**Family Empowerment Scale**									
	Service System Subscale	21	4.3 (4.0-4.6)	18	4.2 (3.9-4.5)	-0.2 (-0.5 to 0.2)	.404	.803	.456
**Dads’ Active Disease Support Scale**									
	Amount score	17	78.1 (61.3-94.8)	16	73.8 (57.5-90.2)	-4.3 (-24.7 to 16.2)	.667	.794	.614
	Helpfulness score	18	70.0 (60.9-79.0)	16	82.3 (72.6-91.9)	12.3 (0.9-23.7)	.036	.211	.161

^a^Estimated marginal mean adjusted for baseline score, severity of chronic kidney disease, ethnicity, and number of parents in family.

^b^Higher scores mean worse outcomes.

**Figure 4:**
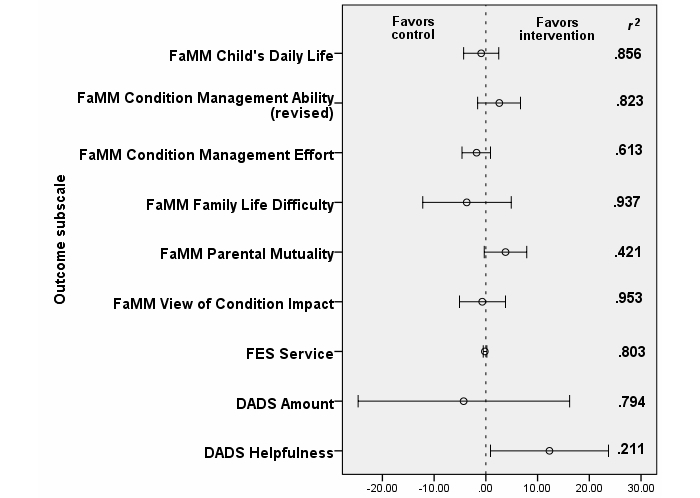
Differences in marginal means between the study groups in terms of whether the difference favored the intervention (OPIS) group or the control group (means adjusted for baseline score, severity of chronic kidney disease, ethnicity, and number of parents in family).

After 20 weeks, parents in the intervention group had an adjusted mean score on the FaMM Condition Management Ability Scale (revised) that was 2.7 points better than that for parents in the control group (95% CI -1.6 to 6.7). The linear mixed model for this outcome was a good fit to the patterns in the data (*r* ^2^=.823). The findings suggest that parents using OPIS tended to perceive themselves to be managing their child’s condition better than parents in the control group perceived themselves to be managing. The adjusted mean for the FaMM Family Life Difficulty Scale was 3.7 points worse in the intervention group (95% CI -4.9 to 12.2, *r* ^2^=.937). This suggested that parents using OPIS tended to perceive having more difficulties with family life due to their child’s condition than parents in the control group perceived themselves to have. Two outcome measures (the FaMM Parental Mutuality Scale and the DADS) applied to families with two parents participating in the study. For the FaMM Parental Mutuality Scale, the adjusted mean at 20 weeks was 3.8 points better (95% CI -0.3 to 7.9, *r* ^2^=.421) for the OPIS group than for the control group. Similarly, the DADS Helpfulness score was 12.3 points better (95% CI 0.9 to 23.7, *r* ^2^=.211) in the intervention group than the control group. That is, using OPIS tended to improve parent mutuality as measured by the FaMM and perceived helpfulness as measured by the DADS. While this feasibility study was not powered to detect significant differences, these four between-group differences seem clinically meaningful, and if these tendencies were to have held up as more parents were recruited to the study, the differences would have been statistically significant. Given the small size of the study sample and even smaller sizes of the treatment groups, these results require examination in a larger and fully powered RCT. Figure 4 presents the differences between the study groups in marginal means at end trial in terms of whether the difference between baseline and week 20 scores on the various outcome measures indicated an advantage to the OPIS group or an advantage to the control group

## Discussion

### Principal Findings

The main findings of this study are that a full-scale RCT of the effectiveness of OPIS is feasible and that OPIS has the potential to beneficially affect self-reported outcomes, including parents’ perceived competence to provide home-based clinical care for children with CKD stage 3-5. In this section, we first address objective 1 by assessing feasibility of a future full-scale RCT of OPIS. Then we address objective 2 by considering the trends in change on study outcome measures.

### Feasibility of Recruitment, Retention, and Data Collection

The results support the feasibility a full-scale RCT. However, in future research recruitment could be improved by examining the potential influences on recruitment to this study. For example, parents could view participating in a study relating to their child’s health care with no promise of benefit to themselves or their child, as adding burden to an already stressful situation. Due to the complex care needs of many children with CKD, in particular those with level 4/5 CKD, the unpredictable nature of individuals’ disease progression and the potential for the child’s condition to deteriorate during the trial, some parents might have declined to participate because of time requirements and duration of commitment.

The researcher recruiting parents and collecting and analyzing data was not a health professional, so not a member of the MDT. Parents were unlikely to feel obliged to agree to the study when approached by the researcher in the way they might have if recruited by a MDT member. We believe it important that the MDT was seen to endorse the research and the researcher. This happened through our strategy of arranging for a professional to introduce the parents to the study during a clinical consultation. This was important because parents often have a long-standing and trusting relationship with members of the MDT and so may have wished to discuss the study with them before making a decision about participation. In future research, more proactive MDT endorsement of the study, such as by referring to OPIS and/or demonstrating it when providing specific information to parents about the child’s condition, could further enhance recruitment.

The study design required that parents be randomly allocated to a treatment group. Some parents who were initially interested in the study could have declined enrollment once they realized there was no certainty of allocation to OPIS. Parents possibly also felt an obligation to express interest in the study to the professional who notified them of the study but later felt able to tell the researcher that they were not interested once they realized that their refusal would not be reported to the MDT. In a future full-scale trial, we can adopt a number of additional strategies to potentially increase recruitment/retention. For example, parents usually have several individual outpatient consultations with members of the MDT at one appointment. We capitalized on the opportunity presented by this as, once the first professional had notified the parents of the study, the researcher then approached them to explain more about what would be involved. Some parents might have been too distracted by their child’s presence and the other pending consultations to be able to give due consideration to the possibility of participating and may have preferred an explanatory telephone call from the researcher at a later date. To enable this, the MDT member who initially informed the parents about the study would also need to ask whether parents would consider either an explanatory phone call from the researcher at a later date or a face-to-face meeting with the researcher in clinic on the same day.

In addition, we could alter the study design so that randomization would be to either the intervention (OPIS) or to a wait-list control group. The wait-list control group could elect to receive the intervention immediately after the post-test assessment [44]. Alternatively, we could release basic OPIS content to both groups (eg, the written, purely informational or technical content) and other OPIS content (eg, the interactive resources to promote clinical caring skills and access to the family-to-family area) to the intervention group only. In addition, data collection, particularly at end of trial may be enhanced as a way to improve retention by offering parents the option to provide data via online means and entirely at their convenience [45].

At the initiation of baseline data collection, the REALM was a useful tool to help the researcher determine whether a parent appeared to struggle with reading. This meant the researcher could alter the data collection strategy accordingly to minimize embarrassment for parents with low health literacy and maximize the quality of the data. The measurements of health literacy were stable from baseline to end of trial, which is understandable since health literacy is a relatively stable construct and OPIS was not intended to improve health literacy. The REALM proved to be difficult for 2 parents due to its large number (66) of items. A recently validated Rapid Estimate of Adult Literacy-Short Form (REALM-SF) comprising only seven items could be more appropriate for a future full-scale RCT of OPIS [46]. However, being asked to complete a health literacy assessment tool for a research project can be embarrassing for parents no matter how sensitively it is delivered. One study found that 40% of patients with low literacy felt ashamed about this [47]. Parents with low health literacy who are responsible for reading and understanding complex information and instructions on how to manage their child’s condition may feel ashamed and also concerned that they might not be able to safely deliver their child’s clinical care. At the same time, they may be embarrassed to tell the MDT that they have health literacy problems in case the professionals would consider them incompetent to enact clinical caring. This fear could have caused great worry for parents who struggled with the REALM. Using the shortened form in future studies would still elicit valuable information while minimizing parental embarrassment and worry. To reduce these problems in the future full-scale RCT, we will administer the REALM-SF at baseline only.

Some potential reasons for attrition in the intervention group are that (1) we adopted a non-directive strategy in that once intervention group parents had received the password and login advice, and (2) we did not direct parents’ use of OPIS as we wished to determine parents’ undirected usage. Parents might have expected more direct and continued engagement with the study team rather than self-directed exploration of OPIS. In addition, parents might have expected more endorsement by MDT members than was possible within the study resources; indeed our qualitative interviews with parents at study exit confirm this [30]. Parents were most likely to have accessed OPIS resources that seemed particularly relevant to their child’s situation and their own health literacy level. Encountering information that provoked uncertainty, fear, and anxiety about their child’s future could then have led to their attrition from the study to avoid further exposure to upsetting information. Furthermore, those who remained in the study through to trial end could have limited their exploration of OPIS, which in turn might have attenuated OPIS effects on study outcomes. In a future study, we will adopt a more engaged and directive approach with participants as a potential means to control attrition and improve retention. For example, we are considering using a combination of targeted delivery of the intervention at regular points in the study period using baseline measurements of personal characteristics and preferences to tailor level of detail and content on an individual basis, at particular time points. We would also interview parents to find out which parts of the OPIS website they found to be most and least relevant.

Retention in research that involves Internet interventions has been identified as a major problem and is now widely recognized as a science of attrition [48]. This body of work has a focus on understanding how the reach of the Internet might increase enrollment of patients at greater risk of attrition, incorporating components into trial design to prevent this effect through understanding patient characteristics associated with higher rates of attrition. While initial attempts to find solutions to the problem of attrition may benefit from investigator intuition and trial-and-error approaches, the area of Internet intervention has advanced to a stage where this delivery modality could benefit from more refined and better specified models, which define the components of individual characteristics, human interaction, and person-to-person support that seem to contribute to adherence. Alternatively, for the intervention group in our study, it may be that some parents did not have time to participate in the trial; they might have been struggling with the clinical care demands meaning that accessing OPIS was an additional demand on their time.

While the numbers dropping out of our study for the different measures were between 4 and 7 parents, patterns of attrition tended to differ between the treatment groups. Although the attrition pattern in the intervention group was inconsistent, those dropping out tended to perceive more problems with family life and more impact caused by their child’s condition at baseline than those intervention group parents who were retained in the study. This finding is consistent with the finding that the scores on these measures were “poorer” in the intervention group than in the control group (see Table 2). This would need to be monitored in a larger study.

### Trends in Change on Study Outcome Measures

To address objective 2, we investigated trends in change on outcomes in a small-scale preliminary RCT in parents of children with CKD stages 3-5. The results suggest that OPIS could improve parents’ ability to manage their child’s condition more than standard care over 20 weeks. For example, after accessing OPIS for 20 weeks parents were less likely to endorse the statement “We have not been able to develop a routine for taking care of our child’s condition”. When children have a chronic condition such as CKD, parents usually assume the roles of care coordinator, clinical expert, and advocate as well as their normal parenting roles. Health care providers are uniquely positioned to assist parents in meeting those challenges, and researchers recommend that they aim to promote parents’ competence and confidence in their child's care through understanding common challenges that parents face, promoting parent-to-parent connections, and building partnerships with parents and their children with clinical needs [49]. Our trial indicates that OPIS appears to address these recommendations. Although the numbers are very small, there seems to be a suggestion that staying in the OPIS group was harder for South Asians, and dropping out from the control group was more common among parents with a child with CKD stage 3 who may be less needy for support and information. These factors may have implications for setting up a full trial, and such attrition would have to be monitored closely. In a full-scale RCT, we would add a measure of self-efficacy, which is the construct that frames the study [50].

The FaMM Parental Mutuality Scale indicated that parents in the intervention group tended to show less decline in satisfaction in working together over time. Reasons for decline in satisfaction should be explored in a larger study. A smaller drop in parental mutuality in intervention group scores on this measure concurred with more desirable change in the DADS Helpfulness scale. A strong correlation between the FaMM Parental Mutuality Scale and the DADS Helpfulness (correlation) subscale suggests that it would not be necessary to include both the FaMM and the DADS in our future full-scale RCT, thus reducing participant burden.

The FaMM View of Condition Impact Scale showed that both groups’ perceived seriousness of the child’s condition tended to show improvement, and more so in the control group as compared to the intervention group.

The FaMM Family Life Difficulty Scale showed that the control group parents appeared to perceive fewer difficulties after 20 weeks, but the intervention group appeared to perceive slightly more. It was not clear whether this was a negative or a positive impact, since OPIS may have elevated parents’ awareness of their caregiving responsibilities. This would need investigating in a larger study using a measure of anxiety as there could be implications for clinical practice.

We also know that adherence to medical recommendations deteriorates over time [51], which has implications for length of participant involvement in the future RCT. Allowing access to OPIS for a longer time period and/or “booster” doses of OPIS to maintain the durability of desired effects are some options to be considered for future studies.

Information management, specifically limiting awareness of information that generates uncertainty, fear, and anxiety has been well established as a typical parental management strategy in the context of life-threatening chronic childhood conditions [52]. However, parents, in particular low-income parents, may be unable to distinguish between high- and low-quality information and may not be confident in using the Internet [24]. This suggests that IHCAs developed between professionals and families and endorsed by professionals for day-to-day use are more likely to reduce anxiety than to increase it. In future research, we could assess parents’ uncertainty tolerance, anxiety, and coping strategies pre- and post OPIS. We could also apply psycho-immunization strategies. Psycho-immunization involves exposing individuals first to relatively benign or generic information and then to increasingly more threatening or relevant information, gradually building up tolerance to uncertainty and controlling the intensity of anxiety and other emotional responses [53].

IHCAs such as OPIS might have the potential to increase fathers’ involvement in disease management if suggestions for refinement and usability issues that we reported elsewhere [54] are addressed in future OPIS development and testing. The DADS Amount scores demonstrate that fathers appreciably contributed to their child’s care; in fact, two participating fathers administered the majority of clinical care because the mother lacked confidence in managing the skills required. The OPIS prototype seems to support and promote collaborative clinical caregiving by fathers and mothers.

### Limitations

A limitation of this study is the low number of participating parents of South Asian descent, despite the relatively high prevalence of CKD in their children. In addition to reasons given by these parents for declining to participate (eg, no time or child transferring to adult care), cultural and language barriers to participation might have been in play. South Asian women, who are often the primary caregivers, can possibly lack knowledge of health risks, have ideas about self-care that differ from those held by white women of European descent, experience language barriers, be subject to the stress of emigration and isolation, be preoccupied with their family’s needs, and not seek access to health promotion programs [55]. Our study was a small, non-powered pilot study, so it could not be all-inclusive given its size, and it was not our intention that the results would be generalizable. Given that children of South Asian descent experience heightened risk for CKD and also parental difficulty in managing CKD, strategies to recruit and retain South Asian parents must be addressed. We did involve South Asian parents in in the Study Steering Group, but their involvement in designing the recruitment strategy will strengthen this aspect of our future research.

The number of eligible children being cared for at the site during the study period was less than anticipated, and we did not achieve the target of data from 30 parents per group at trial end. The future full-scale RCT could recruit parents from multiple sites, with the intervention being delivered via the Web as in the feasibility study. Overall, the data obtained from our 51 participants were highly informative.

### Conclusion

Our results indicate that a full-scale RCT of OPIS is feasible. Furthermore, being in the intervention group improved parents’ perceived management ability to a greater extent than usual care over 20 weeks. Specifically, the FaMM Condition Management Ability Scale appeared to show beneficial change when reinforced by accessing OPIS. A full-scale trial of OPIS is indicated that would include a shorter measurement of health literacy and additional measurements of self-efficacy and anxiety, with an embedded qualitative component to investigate reasons underlying changes in outcome scores.

## Abbreviations

CKD: chronic kidney disease
DADS: Dads Active Disease Support scale
FaMM: Family Management Measure
FES: Family Empowerment Scale
HON: Health on the Net
IHCA: interactive health communication application
MDT: multidisciplinary team
NHS: National Health Service
OPIS: Online Parent Information and Support
REALM: Rapid Estimate of Health Literacy
REC: Research Ethics Committee
